# Mislocalization after inhibition of saccadic adaptation

**DOI:** 10.1167/jov.22.8.3

**Published:** 2022-07-14

**Authors:** Frauke Heins, Markus Lappe

**Affiliations:** 1 Institute for Psychology and Otto-Creutzfeldt Center for Cognitive and Behavioral Neuroscience, University of Muenster, Muenster, Germany; 2 Institute for Psychology and Otto-Creutzfeldt Center for Cognitive and Behavioral Neuroscience, University of Muenster, Muenster, Germany

**Keywords:** saccade adaptation, eye movements, visual perception, sensorimotor adaptation

## Abstract

Saccadic eye movements are often imprecise and result in an error between expected and actual retinal target location after the saccade. Repeated experience of this error produces changes in saccade amplitude to reduce the error and concomitant changes in apparent visual location. We investigated the relationship between these two plastic processes in a series of experiments. Following a recent paradigm of inhibition of saccadic adaptation, in which participants are instructed to look at the initial target position and to continue to look at that position even if the target were to move again, our participants nevertheless perceived a visual probe presented near the saccade target to be shifted in direction of the target error. The location percept of the target gradually shifted and diverged over time from the executed saccade. Our findings indicate that changes in perceived location can be the same even when changes in saccade amplitude differ according to instruction and can develop even when the amplitude of the saccades executed during the adaptation procedure does not change. There are two possible explanations for this divergence between the adaptation states of saccade amplitude and perceived location. Either the intrasaccadic target step might trigger updating of the association between pre- and post-saccadic target positions, causing the localization shift, or the saccade motor command adjusts together with the perceived location at a common adaptation site, downstream from which voluntary control is exerted upon the executed eye movement only.

## Introduction

We perform more than 100,000 saccadic eye movements every day to look at targets of interest. These eye movements are so fast that visual information about the accuracy of the saccade becomes available only after the saccade has ended. An oculomotor learning process, termed *saccadic adaptation*, evaluates saccade error and adjusts motor performance of future similar saccades to maintain accuracy despite injury, disease (Abel, Schmidt, Dell'Osso, & Daroff, 1978; Kommerell, Olivier, & Theopold, 1976; Optican & Robinson, 1980), fatigue (Prsa & Thier, 2011), or aging (Munoz, Broughton, Goldring, & Armstrong, 1998; Warabi, Kase, & Kato, 1984). Saccadic adaptation is studied in the double-step paradigm (McLaughlin, 1967) by stepping the saccade target once the saccade has been initiated. The target step, unless it is very large with respect to the saccadic amplitude, usually goes unnoticed due to saccadic suppression of displacement (Bridgeman, Hendry, & Stark, 1975; Deubel, Wolf, & Hauske, 1986; Stark, Kong, Schwartz, & Hendry, 1976). The target step introduces a post-saccadic error because the visual image of the target is not in the expected location after the saccade. If this procedure is repeated over several trials, saccade amplitude adapts and the magnitude of the post-saccadic error is gradually reduced (McLaughlin, 1967).

Saccadic adaptation is known to affect also visual space perception. The apparent location of visual targets shifts in the adaptation direction (Awater, Burr, Lappe, Morrone, & Goldberg, 2005; Bahcall & Kowler, 1999; Bruno & Morrone, 2007). The effect is strongest for objects presented in the area near the original target position (Awater et al., 2005; Bruno & Morrone, 2007) and resembles that of the adaptation field for saccades (Collins, Doré-Mazars, & Lappe, 2007; Schnier, Zimmermann, & Lappe, 2010). The adapted state of a saccade toward a position within the adaptation field is correlated with the size of the perceptual mislocalization at that position (Collins et al., 2007; Schnier et al., 2010). This implies that saccade targeting and perceptual localization share a common coordinate system (Collins et al., 2007; Zimmermann & Lappe, 2009, 2010).

The close link between saccadic adaptation and spatial perception indicates that either adaptive changes to perceived location follow adaptation of saccade amplitude or adaptive changes to perceived location contribute to the development of amplitude adaptation. To investigate this question, we employed a recently developed paradigm of inhibition of saccadic adaptation (Heins, Meermeier, & Lappe, 2019). In this paradigm, participants are exposed to the same sequence of events as in regular double-step saccadic adaptation, but they are instructed to saccade only to the initial target location and remain there even if the target were to move again. In this paradigm, participants were able to inhibit any changes to saccadic amplitude for outward target steps and to strongly reduce changes to saccade amplitude for inward target steps (Heins et al., 2019). The successful inhibition of adaptive changes to the saccade amplitude following outward adaptation went along with an increase in saccadic latency, presumably reflecting an effort to suppress a reflexive saccade to the target and reprogram the saccade. In the present study, we combine our paradigm of inhibition of saccadic adaptation with a localization task. In a series of experiments, following inhibition of saccade amplitude adaptation, participants were asked to localize either a pre-saccadically presented localization probe or the saccade target itself after saccade execution.

## Experiment 1—adaptation versus inhibition

In our first experiment, we investigated saccade amplitude and localization following both the inhibition and adaptation instruction for inward and outward target displacement in identical double-step sequences. We determined the change in both perceived object location and saccade amplitude and compared the effect of the instruction to look at the initial target position and to keep gaze there with the instruction to look at the target and to follow to the target to its final position.

### Method

#### Sample

The sample consisted of 16 participants (9 female) aged between 18 and 46 years (*M* = 27.69, *SD* = 6.97). All participants had normal or corrected-to-normal vision. All but one of the participants were right-handed. All participants were recruited from the Department of Psychology of the University of Muenster and gave their informed consent in written form.

#### Experimental setup

Experimental procedures were approved by the Ethics Committee of the Department of Psychology and Sports Science of the University of Muenster. The experiment was conducted in a dimly lit room in the Institute of Psychology of the University of Muenster. The participants were seated at a 69-cm distance of an Eizo FlexScan 22-in. monitor (Eizo, Hakusan, Japan) with a screen resolution of 1152 × 870 pixels at a frame rate of 75 Hz. The eye position was measured using an Eyelink 1000 eye tracker (SR Research, Ontario, Canada) at a sampling frequency of 1000 Hz. The experimental code was written in MATLAB (R2018a; The MathWorks), and for stimulus presentation, we used the Psychophysics Toolbox extension (Brainard, 1997; Kleiner et al., 2007). Viewing was binocular, but only the right eye was recorded. A custom-developed combined chin-forehead-rest was used to ensure a stable head position during the recording session.

#### Stimuli and procedure

**Figure 1.**
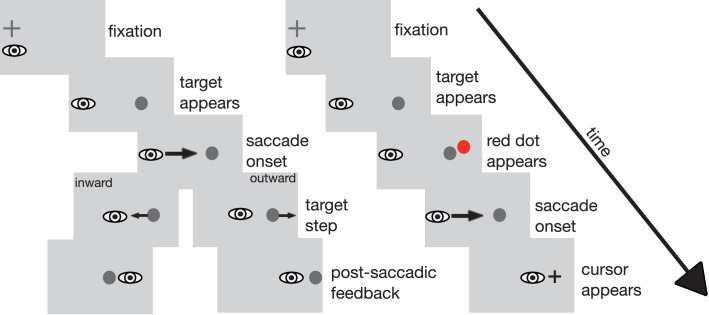
Trial layout for double-step trials (left) with inward and outward target steps and for localization trials (right) in Experiment 1. In the double-step trials, participants first looked at a fixation cross. Then, the fixation cross turned off and the saccade target appeared 12 deg to the right. Upon saccade onset, the target was stepped, depending on condition, either in the inward or the outward direction, leading to a post-saccadic error after saccade landing. The target remained visible at the stepped location for 500 to 1000 msec. In the localization trials, participants looked at the fixation cross until the saccade target appeared. Fifty milliseconds later (i.e., during the saccade latency period), a red probe dot was displayed for two frames (27 msec). After saccade onset, the saccade target was turned off and the cursor appeared. After the saccade, participants indicated the perceived position of the red probe dot with the cursor. Stimuli are not drawn true to scale.

Stimuli were presented on a mid-gray background. A black fixation cross (0.6 × 0.6 deg) was displayed on the left side of the screen. Its position varied between trials on the horizontal (up to 2 deg left or right) and the vertical axis (up to 1 deg up- or downward) in a counterbalanced manner to avoid predictability. Before a trial started, the participant's eye position had to be within 3 deg of the fixation cross for a random time interval of 700 to 1300 msec. At the start of the trial, the fixation cross was removed and the target appeared. The target was a dark gray dot of 0.5 deg diameter presented 12 deg to the right of the fixation cross. Subsequent events depended on the type of trial. Regular double-step trials, localization trials, and target-off trials were used. In regular double-step trials, when the eye had moved 3 deg toward the target and eye velocity exceeded 138 deg/sec, the intrasaccadic target step was triggered. The direction of the step remained the same during a recording session and depended on the current experimental condition. The size of the intrasaccadic target-step varied randomly from 20%, 30%, or 40% of the original saccade amplitude (12 deg), which corresponds to an absolute size of the target displacement of 2.4, 3.6, or 4.8 deg. This variation was included to avoid predictability of the target-step size in the inhibition conditions. The target remained visible at the stepped position for 500 to 1000 msec until the screen went blank and the next trial began. In the target-off trials, the target was extinguished upon saccade detection. In the localization trials, a red probe dot appeared for two video frames (27 msec) starting 50 msec after presentation of the target and before the saccade started. The dot could appear on the horizontal target position or 1 deg to the left or right of that location. Its vertical position was 2 deg above the saccade target. Then, when the eye had moved 3 deg toward the target and the eye velocity exceeded 138 deg/sec, the target was extinguished and a cross-hair mouse pointer appeared at the screen position that participants had clicked on in the previous localization trial. After the saccade, participants indicated the perceived position of the red dot by clicking at the respective position on the screen with the mouse pointer. Thereafter, the pointer disappeared. The sequence of events in double-step and localization trials is depicted in Figure 1.

#### Conditions

To investigate localization after inhibition of adaptive changes to saccade amplitude compared to regular adaptation in identical double-step sequences, we measured four experimental conditions following Heins et al. (2019). For the inhibition conditions, participants were instructed to look at the fixation cross, saccade toward the target as soon as it appeared, and, irrespective of any possible further movement of the target, continue to look at the same position. In the adaptation conditions, participants were told to look at the fixation cross, saccade toward the target, and follow any further target movements to look at its final position. These instructions necessarily alerted participants that the target might move more than once. However, awareness of the second movement of the target does not affect adaptation since participants who notice the intrasaccadic target step show the same amount of adaptation as unaware participants (Frens & van Opstal, 1994; Heins et al., 2019).

The experiment consisted of four recording sessions per participant, each lasting approximately 20 min and testing different combinations of target step direction and instruction: outward inhibition, inward inhibition, outward adaptation, and inward adaptation. In each recording session, one of the four experimental conditions was recorded. As it was vital for our results to avoid adaptation carryover from one recording session to another, the minimum time interval between two recording sessions was 7 days (Alahyane & Pélisson, 2005). Participants first completed both inhibition conditions before they participated in the adaptation conditions. This was also done in order to avoid any possible carryover from regular adaptation to the inhibition conditions. The order of adaptation direction was counterbalanced between participants.

Each session began with 20 pre-adaptation localization trials, which were followed by 20 pre-adaptation target-off trials. The subsequent double-step phase consisted of 150 double-step trials, during which the target was stepped. The double-step phase was followed by 20 post-adaptation localization and then 20 post-adaptation target-off trials. Every 60 trials, the experiment paused and an audio file was played that contained a verbal instruction for the respective condition and thus reminded the participant of the task. The participants could freely choose whether they wanted to close their eyes or keep them open during the 25-sec break. A tone indicated that the experiment continued.

#### Data analysis

Trials with primary saccades of an amplitude of less than 6 or more than 18 deg or with latencies of less than 100 or more than 400 msec were discarded (8.10%). To avoid contamination by saccadic compression (Lappe et al., 2000; Ross, Morrone, & Burr, 1997), localization trials in which the probe (red dot) was presented less than 90 msec prior to saccade onset or in which the participant had indicated that he had not seen the red dot were also discarded (9.14%).

Data analysis was conducted with MATLAB (R2018a; The MathWorks) and R (version 4.0.2; R Development Core Team). The amplitude gain change was used to evaluate the magnitude of adaptation. It was calculated with the following equation:

(1) $$GC=\frac{A-{\bar{A}}_{pre}}{{\bar{A}}_{pre}}\times 100$$

In this calculation, the average pre-adaptation amplitude, which was measured during the pre-adaptation target-off trials, was subtracted from the amplitude made in the respective trials. This difference then was divided through the average pre-adaptation amplitude and multiplied by 100 to obtain the change in saccadic gain in percent (Panouillères et al., 2009).

Localization change was evaluated in an identical manner by calculating localization gain change:

(2) $$LC=\frac{L-{\bar{L}}_{pre}}{{\bar{L}}_{pre}}\times 100$$

Here, *L* refers to the horizontal distance of the perceived location of the red probe dot from the fixation point, similar to the amplitude used for the saccade.

We investigated the effect of instruction and double-step direction on both saccade characteristics and localization judgment using repeated-measures analyses of variance (ANOVAs). If the normality assumption was violated, a nonparametric repeated-measures ANOVA was computed using the aligned rank transform (Wobbrock, Findlater, Gergle, & Higgins, 2011). Differences between conditions were assessed using paired and unpaired *t* tests with an alpha level of 0.05. If the requirements for parametric statistics were not met, the Wilcoxon signed-rank test was performed. The *p* values were corrected with the Bonferroni–Holm procedure. In cases where the equivalence of two conditions was to be demonstrated, Bayesian hypothesis tests were performed to determine evidence for or against similarity of the respective measures. We computed Bayes factors using JASP (version 0.13.0; JASP Team) with a range of prior widths for a robust analysis.

### Results

**Figure 2.**
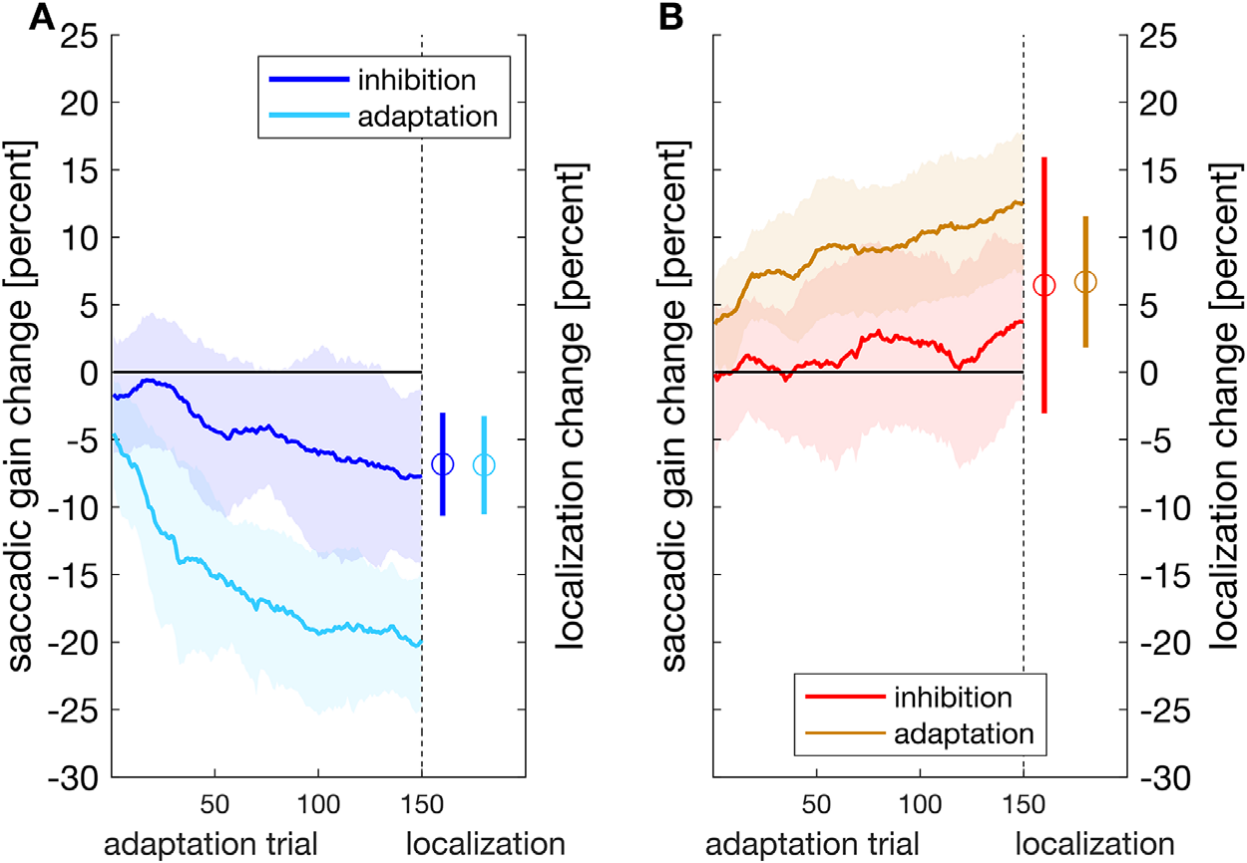
Moving average (window size 20 trials) of the saccadic gain change during the double-step procedure and mean localization change during the subsequent localization trials for inward (A) and outward target steps (B) for the inhibition instruction and for the adaptation instruction. The shaded areas as well as the vertical bars indicate standard deviations.

First, we checked the effectiveness of our instruction and, therefore, whether adaptive changes to saccade amplitude occurred following the adaptation instruction and whether they were inhibited following the inhibition instruction. In the adaptation conditions, we found learning curves as typically described in literature (Deubel et al., 1986; Ethier et al., 2008; Panouillères et al., 2009) for both inward (Figure 2A, turquoise curve) and outward (Figure 2B, yellow curve) target steps. ln the inhibition conditions, the change in saccadic gain was much smaller consistent with our previous study on inhibition of saccadic adaptation (Heins et al., 2019). Time courses for gain changes in the inhibition conditions are shown in Figure 2 (left) for inward target steps (blue curve) and in Figure 2 (right) for outward target steps (red curve). For quantitative analysis, we computed saccadic gain change for the late-adaptation trials (trials 171:190), that is, immediately before the localization trials. The step direction had a significant effect on saccadic gain change (*F*(1, 15) = 171.472, *p* < 0.001), whereas the main effect of instruction was not significant (*F*(1, 15) = 0.595, *p* = 0.453). This is not surprising because the differences in gain change following the inhibition instruction and the adaptation instruction are canceled out by the different signs of gain change after inward and outward target steps. The interaction of step direction and instruction was significant (*F*(1, 15) = 71.580, *p* < 0.001). The average change in saccadic gain was smaller for the inhibition conditions than for the adaptation conditions for both inward (inhibition: *M* = –7.82%, *SD* = 5.91%; adaptation: *M* = – 19.74%, *SD* = 4.38%; *t*(15) = 6.853, *p* < 0.001, one-sided *t* test) and outward (inhibition: *M* = +2.93%, *SD* = 6.99%; adaptation: *M* = +12.03%, *SD* = 4.99%; *t*(15) = –4.442; *p* < 0.001, one-sided *t* test) target steps. This implies that the inhibition instruction led to a weaker change in saccadic gain for both inward and outward target displacement compared to regular adaptation, as expected.

Separate tests against zero in the inhibition conditions showed that gain change deviated from zero (*t*(15) = –5.291, *p* < 0.001, one-sided *t* test) following inward target steps. For outward target steps, gain change was not different from zero (*t*(15) = 1.676, *p* = 0.057, one-sided *t* test; Bayes factors *BF*_01_ = 1.241 [1.538, 2.000]). In the adaptation conditions, saccadic gain change deviated from zero both for inward (*p* < 0.001, one-sided Wilcoxon signed-rank test) and outward target steps (*t*(15) = 9.649, *p* < 0.001, one-sided *t* test).

We also analyzed how much saccadic gain change was retained in the target-off trials that were performed after the localization trials, as adaptation usually does not fully transfer from double-step trials to trials without the respective target displacement (Alahyane et al., 2007; Frens & van Opstal, 1994). As in the late-adaptation trials, the step direction had a significant influence on saccadic gain change (*F*(1, 15) = 269.958, *p* < 0.001), whereas the main effect of direction was not significant (*F*(1, 15) = 0.664, *p* = 0.428). The interaction was significant (*F*(1, 15) = 17.751, *p* < 0.001). The average gain change in the post-adaptation trials was –8.53% (*SD* = 4.81%) in the inward inhibition, –12.70% (*SD* = 4.04%) in the inward adaptation, +4.35% (*SD* = 5.14%) in the outward inhibition, and +6.86% (*SD* = 3.72%) in the outward adaptation condition. For the instruction to adapt, gain change was smaller than zero for inward target steps (*t*(15) = –12.575, *p* < 0.001, one-sided *t* test) and larger than zero for outward target steps (*t*(15) = 7.387, *p* < 0.001). Following the inhibition instruction, gain change was smaller than zero for inward target steps (*t*(15) = –7.093, *p* < 0.001, one-sided *t* test), as before, but, unlike in the late-adaptation trials, gain change exceeded zero for outward target steps (*t*(15) = 3.387, *p* = 0.004, one-sided *t* test). This might indicate that in the target-off trials at the end of the experiment, some of the inhibition that took place in the outward inhibition condition was discontinued.

We then analyzed the localization change in the different conditions. Strikingly, localization changed significantly in the inhibition conditions and, moreover, in much the same manner as in the adaptation conditions. Average values of localization gain change are shown in Figure 2. Following inward target displacement, the average localization change was –6.83% (*SD* = 3.83%) for the inhibition instruction and –6.89% (*SD* = 3.63%) for the adaptation instruction. Following outward target steps, the average localization change was 6.44% (*SD* = 9.50%) for the inhibition instruction and 6.70% (*SD* = 4.86%) for the adaptation instruction. The direction of the target step had a significant influence on the localization change (*F*(1, 15) = 118.311, *p* < 0.001), whereas the instruction had not (*F*(1, 15) = 0.370, *p* = 0.552). The interaction of step direction and instruction was not significant (*F*(1, 15) = 0.002, *p* = 0.961). The *t* tests verified that the shift in localization judgment that developed in the inhibition condition was significant for both inward (*t*(15) = – 7.136, *p* < 0.001, one-sided *t* test) and outward target steps (*p* = 0.004, one-sided Wilcoxon signed-rank test) and, more important, was of equal magnitude for the inhibition and adaptation instruction (inward: *t*(15) = 0.056, *p* = 0.955, two-sided *t* test, *BF*_01_ = 2.924 [3.846, 5.181]; outward: *t*(15) = –0.138, *p* = 0.892, two-sided *t* test, *BF*_01_ = 2.967 [3.906, 5.263]). These results imply that a change in perceived localization in response to the exposure to double-step trials even occurs when adaptation of saccade amplitude is significantly attenuated due to inhibition instruction.

**Figure 3.**
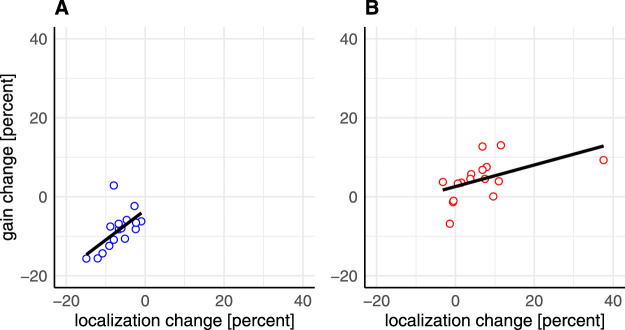
Correlation between localization change and saccade gain change in the inward inhibition (A) and the outward inhibition (B) condition during post-adaptation trials.

We also investigated the relation between localization change and gain change during late- and post-adaptation trials in each experimental condition. Late-adaptation trials showed no significant correlations for any condition (inward: inhibition: *r* = 0.389, *p* = 0.136; adaptation: *r* = 0.347, *p* = 0.188; outward: inhibition: *r* = 0.480, *p* = 0.062; adaptation: *r* = 0.309, *p* = 0.244), and neither did the post-adaptation trials following the adaptation instruction (inward: *r* = 0.366, *p* = 0.163; outward: *r* = 0.453, *p* = 0.080). For the inhibition instruction, however, the post-adaptation trials, during which the intrasaccadic manipulation was no longer applied, showed a significant correlation between localization change and gain change for both inward (*r* = 0.601, *p* = 0.014) and outward (*r* = 0.679, *p* = 0.005) target steps (Figure 3).

**Figure 4.**
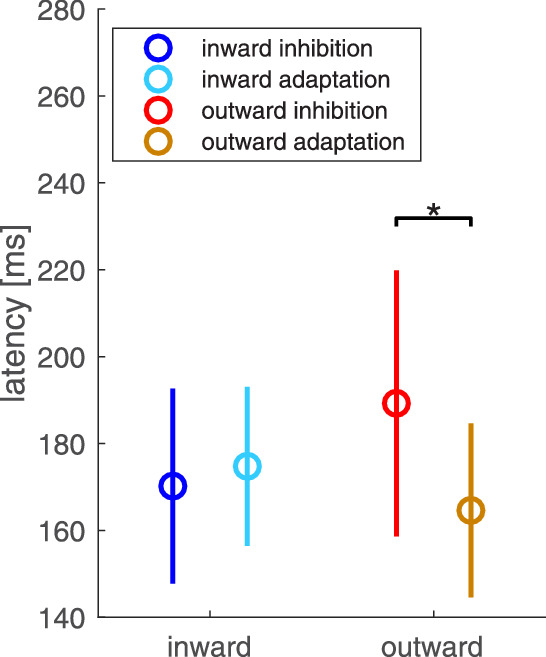
Mean saccadic latency for outward and inward target steps and both the inhibition and adaptation instruction, depicted for the late-adaptation trials. The bars indicate standard deviations.

Previous work on inhibition of adaptation had shown a distinct increase of saccade latency when participants were instructed to inhibit outward adaptation (Heins et al., 2019). We therefore investigated the effect of the instructions on saccadic latency (Figure 4). During the late-adaptation trials, the average latency was 170.20 msec (*SD* = 22.46 msec) in the inward inhibition condition, 174.76 msec (*SD* = 18.30 msec) in the inward adaptation condition, 189.25 msec (*SD* = 30.62 msec) in the outward inhibition condition, and 164.61 msec (*SD* = 20.02 msec) in the outward adaptation condition. For inward target steps, there was no significant difference in saccadic latency between the two instructions (*t*(15) = 0.850, *p* = 0.409, two-sided *t* test; *BF*_01_ = 2.857 [3.771, 5.114]), but for outward target steps, the inhibition instruction led to longer latencies than the adaptation instruction (*p* = 0.013, two-sided Wilcoxon signed-rank test). This confirms our previous report (Heins et al., 2019).

During the target-off trials at the end of the experiment, saccadic latency was 179.63 msec (*SD* = 26.05 msec) in the inward inhibition condition and 168.65 msec (*SD* = 22.69 msec) in the inward adaptation condition. For outward target steps, mean saccadic latency was 178.45 msec (*SD* = 22.30 msec) following the inhibition instruction and 166.22 msec (*SD* = 26.98 msec) following the adaptation instruction. No difference in saccadic latency occurred due to the instruction, neither for inward target steps (*t*(15)= 2.369, *p* = 0.095, two-sided *t* test; *BF*_01_ = 2.15 [1.855, 1.499]) nor for outward target steps (*t*(15) = 2.369, *p* = 0.095, two-sided *t* test; *BF*_01_ = 2.15 [1.855, 1.499]). These findings imply that a prolongation of saccade latency occurred during the outward inhibition condition but less so in the later target-off trials that had shown some reduction of inhibition in the saccadic gain changes.

### Discussion

Experiment 1 showed that the localization shift that followed the repeated intrasaccadic shift of the target stimulus occurred similarly when participants were instructed to look at the initial target position and to continue to look at this position even if the target were to move again and when participants were instructed to look at the target and to follow it to its final position, even though the inhibition instruction produced much less change to saccade amplitude than the adaptation instruction. Following the inhibition instruction, there was residual adaptation for inward target steps, whereas saccadic gain change for outward target steps did not exceed zero significantly. This is consistent with our previous finding that inhibition of outward adaptation prevents significant changes in saccadic gain while inhibition of inward adaptation retains some residual adaptation (Heins et al., 2019). Differences between inward and outward adaptation are not uncommon and have been reported for many different aspects of adaptation (Ethier et al., 2008; Miller et al., 1981; Panouillères et al., 2009; Straube & Deubel, 1995; Straube et al., 1997). They are taken as evidence of partly different mechanisms (Pélisson, Alahyane, Panouillères, & Tilikete, 2010). We will come back to this in the general discussion. In the post-adaptation trials, during which the intrasaccadic manipulation was removed, the pattern of results was different. Following the inhibition instruction, participants now also showed significant gain change for outward target steps. This indicates that some of the inhibition was lost during the target-off trials. This is remarkable, given that usually saccadic gain change is less pronounced during post-adaptation than late-adaptation trials as adaptation typically does not fully transfer from trials with the intrasaccadic target step to trials without the manipulation (Alahyane et al., 2007; Frens & van Opstal, 1994).

The main result of Experiment 1 is that the change in perceived localization occurred similarly when adaptation of saccadic amplitude was significantly attenuated due to the inhibition instruction than when saccade amplitude adapted strongly due to the adaptation instruction. This indicates that the magnitude of the localization shift is not directly related to the amplitude of the executed saccade. Instead, it either must have been acquired from the transsaccadic target shift directly or the adaptation of the saccade took place latently but was not overtly shown. In that view, inhibition of saccadic adaptation might involve both a suppression of a reflexive saccade and the preservation of the movement vector specifying a saccade toward the initial target position, while simultaneously a new association between pre- and post-saccadic visual target positions is learned latently during the adaptation procedure. This resembles the latent learning we observed in another recent study (Heins & Lappe, 2022). In that study, participants had to saccade toward a specified object within an object array. Although the object array was shifted against saccade direction during the eye movement, participants needed to maintain a stable saccade gain in order to look at the target object and achieve the task goal. Participants managed to actively control their oculomotor behavior and keep the saccade gain stable for as long as the intrasaccadic manipulation was applied. When the intrasaccadic manipulation was removed, however, participants exhibited a significant aftereffect (i.e., a significant gain change in direction of the array shift), showing that adaptation was learned but not expressed because it would have reduced task performance. Our current results may point in the same direction. Perhaps the localization shift developed because of the continuing intrasaccadic manipulation during the adaptation phase, but the inhibition task required active, volitional control to suppress the execution of an adapted saccade in order to comply with the instruction. This also ties in with our current result that during late-adaptation trials with outward target steps, saccade latencies were longer following the inhibition instruction than following the adaptation instruction. Once the intrasaccadic manipulation was removed, the effect of instruction on saccadic latency was no longer substantial and simultaneously a significant gain change emerged. It seemed that when participants no longer actively maintained execution of a 12-deg saccade to the initial target position, the adapted state of the saccade amplitude, latently acquired during the double-step procedure, became manifest. This is also consistent with the significant correlations between localization change and gain change following the inhibition instruction in the post-adaptation trials only. We suggest that when participants gave up their effort to perform a saccade to the initial target position, the magnitude of adaptation of the executed saccade amplitude was related to the changes in perceived location, unlike in the late-adaptation trials.

## Experiment 2—time course

The design of Experiment 1 did not allow us to draw conclusions about the time course of the development of the localization shift, and because of the blockwise arrangement of localization, target-off, and adaptation trials, we also could not rule out the possibility that our participants planned or executed their saccades differently in localization and double-step trials. Thus, in Experiment 2, the time course of adaptation of saccade amplitude and localization were assessed throughout the experiment. We interspersed regular double-step trials randomly with target-off localization trials to avoid pre-saccadic cues to the type of task and, thus, to prevent participants from planning or executing their saccade differently in localization trials.

### Method

#### Sample

The sample consisted of 18 participants (11 female) aged between 19 and 46 years (*M* = 27.17, *SD* = 6.66). All participants had normal or corrected-to-normal vision and were right-handed. All participants were recruited from the Institute of Psychology of the University of Muenster and gave their informed consent in written form. Eight participants had also participated in Experiment 1. Two participants had to be excluded from data analysis. One of them did not follow the instruction to saccade toward the target as soon as it appeared and instead delayed saccade onset, presumably with the intention to capture any possible further movement of the target before initiating the saccade. This led to saccade latencies above 400 msec (our predefined exclusion criterion) in more than 50% of the trials. The other participant experienced difficulties performing accurate eye movements to any stimulus on the screen, which produced huge variation in saccadic amplitude and violation of our inclusion criteria for saccade amplitudes in more than 50% of the trials.

#### Experimental setup and stimuli

**Figure 5.**
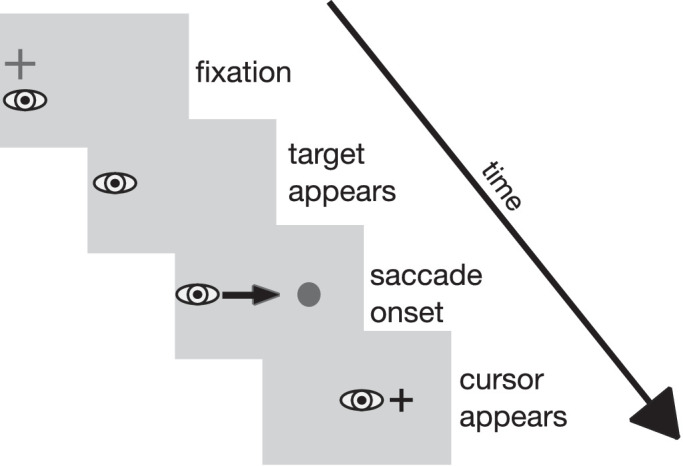
Trial layout for the localization trials in Experiment 2. Participants first looked at a fixation cross. Then, the fixation cross was turned off and the saccade target appeared. After saccade onset, the saccade target was turned off and the cursor appeared. After the saccade, the participants indicated the perceived position of the target stimulus with the cursor. Stimuli are not drawn true to scale.

The experimental setup of Experiment 2 was the same as in Experiment 1. The stimuli resemble those in Experiment 1, except that during localization trials, the target disappeared during the saccade and no red dot was presented. Instead, the participants had to indicate the perceived location of the saccade target after the saccade. The sequence of events in a localization trial is depicted in Figure 5.

#### Conditions

The experiment consisted of two recording sessions per participant, outward inhibition and inward inhibition, each lasting approximately 25 min. To avoid adaptation carryover from one recording session to another, the minimum time period between the two recording sessions was 7 days (Alahyane & Pélisson, 2005). The order of direction (inward/outward) was counterbalanced between participants.

Every session comprised 300 trials. These consisted of 246 double-step trials and 54 localization trials. The localization trials were randomly arranged with the condition that a minimum of three and a maximum of six double-step trials were run in between. The number of double-step trials varied randomly between two and six to avoid predictability.

As in Experiment 1, every 60 trials, the experiment paused and the audio file containing the instruction to continue to look at the initial target position was played.

#### Data analysis

As there were no designated pre- and post-adaptation trials, we assessed the saccadic gain change and the localization shift as a continuous time series to find out whether and at which time in the experiment our dependent variable deviated from a baseline value. We used the SMART method (van Leeuwen, Smeets, & Belopolsky, 2019) to apply cluster-based permutation testing to behavioral data with one data point per trial. The SMART method involves generating a temporally smoothed time series of the data for each participant, constructing a weighted average time series across all participants and performing cluster-based permutation testing. First, the dependent variable was smoothed with a moving Gaussian window (σ = 10 trials for saccadic gain change; σ = 5 trials for localization change). Weighted averaging across participants then resulted in a smoothed average time series for each of the two conditions. A weighted *t* test was calculated for each time point of the smoothed data. Since the dependent variable in a given trial of the experiment was not independent of its value in a preceding or succeeding trial, clusters of temporally adjacent trials emerged. Clusters of significant differences were defined as two or more consecutive time points with *p* < 0.05 and cluster strength as the sum of *t* values in the cluster. For the permutation, the condition labels were then randomly shuffled. For each permutation, the sum of *t* values for the largest cluster entered the permutation distribution. The procedure was repeated 10.000 times. To identify significant clusters in the nonpermuted data, cluster strength was compared to the permutation distribution, and any cluster in the original data with a cluster strength exceeding the 95th percentile was considered significant. For significant clusters, we report the cluster strength (*t*) along with the critical *t* value (*t*_crit_) and the corresponding *p* value.

Exclusion criteria for saccades and localization judgments were the same as in Experiment 1. A total of 13.25% of saccades had to be discarded under these criteria, as well as 4.69% of the localization judgments.

Gain change and localization change were calculated as dependent measures as in Experiment 1. In Experiment 2, however, we had no designated pre- and post-adaptation trials. Thus, the average localization amplitude in the first 10 localization trials was taken as baseline and subtracted from the localization amplitude in the respective trials. This difference then was divided through the average localization amplitude in the first 10 localization trials and multiplied by 100 to obtain the change in localization in percent.

Unlike Experiment 1, Experiment 2 allowed a direct comparison between saccade landing location and perceived target location in individual trials. For this analysis, we calculated the apparent visual error as the difference between the reported target location and the landing point of the saccade.

#### Results and discussion

**Figure 6.**
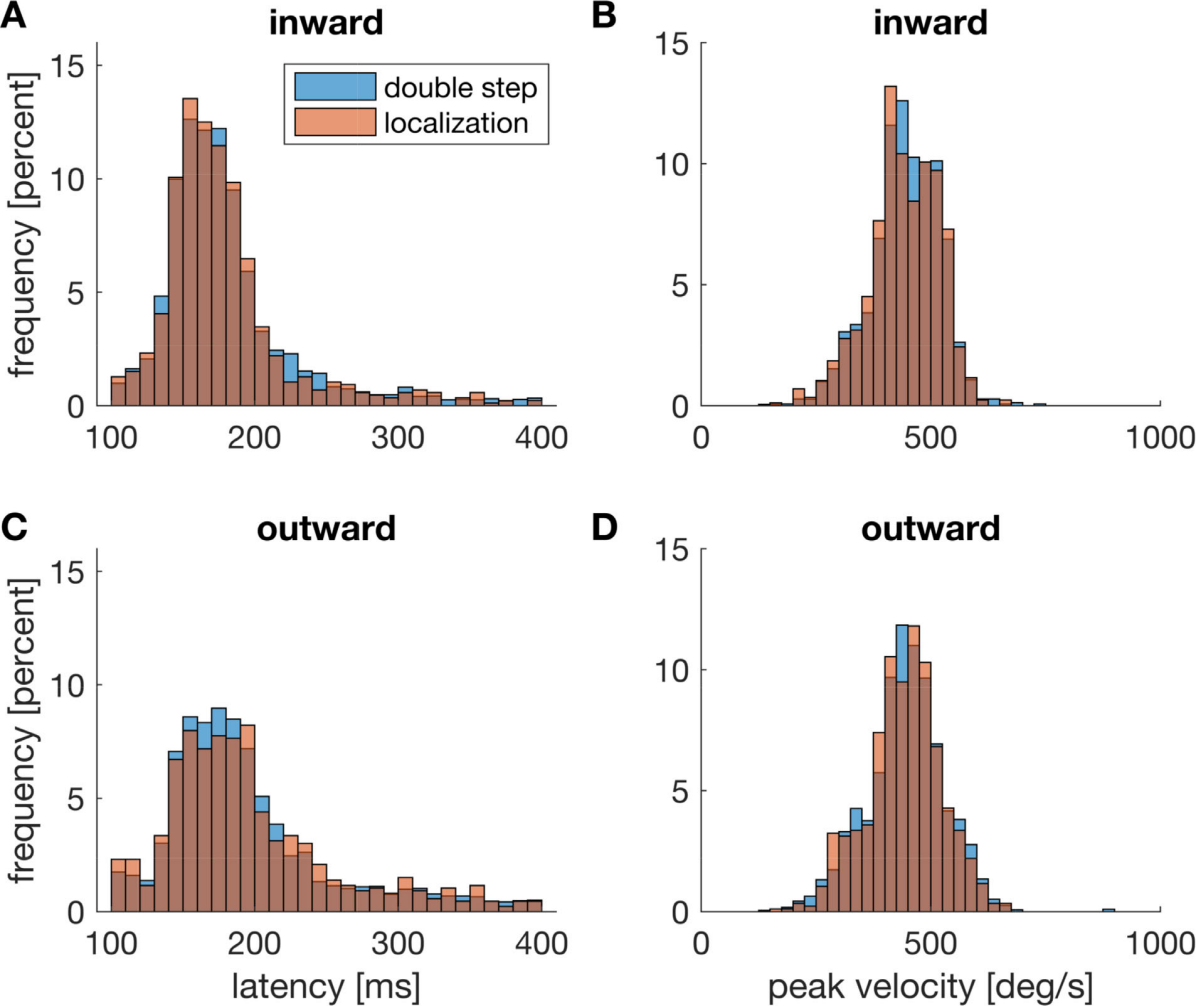
Distribution of saccadic latency (left) for inward (A) and outward (C) target displacement. Right: distribution of peak velocity for inward (B) and outward (D) target steps. Double-step trials are depicted in blue, localization trials in brown.

To find out whether the design ensured that saccades were planned and executed in the same way in double-step and localization trials, we first examined whether saccadic latency and peak velocity differed between the different trial types. Figure 6 depicts the distributions of saccadic latency and peak velocity in the double-step and localization trials. For inward target steps, mean saccadic latency was 178.28 msec (*SD* = 16.77 msec) in localization and 179.08 msec (*SD* = 17.94 msec) in double-step trials. The difference was not significant (*t*(15) = –0.665, *p* = 0.515, two-sided *t* test; *BF*_01_ = 3.226 [4.292, 5.848]). For outward target steps, mean saccadic latency was 196.67 msec (*SD* = 32.29 msec) in localization and 195.15 msec (*SD* = 30.81 msec) in double-step trials and also not significantly different from each other (*t*(15) = 0.467, *p* = 0.647, two-sided *t* test; *BF*_01_= 3.559 [4.762, 6.536]). Peak velocity for inward target steps did not differ significantly between double-step (*M* = 445.12 deg/sec, *SD* = 53.96 deg/sec) and localization trials (443.47 deg/sec, *SD* = 56.57 deg/s; *t*(15) = –1.140, *p* = 0.272, two-sided *t* test; *BF*_01_ = 2.247 [2.907, 3.891]). Peak velocity for outward target steps also did not differ between double-step (*M* = 440.29 deg/sec, *SD* = 69.87 deg/sec) and localization trials (*M* = 436.41 deg/sec, *SD* = 71.28 deg/sec; *p* = 0.231, Wilcoxon signed-rank test; *BF*_01_ = 2.318). These results indicate that saccades of the same type were performed for both trial types, as expected from the absence of any pre-saccadic cue.

**Figure 7.**
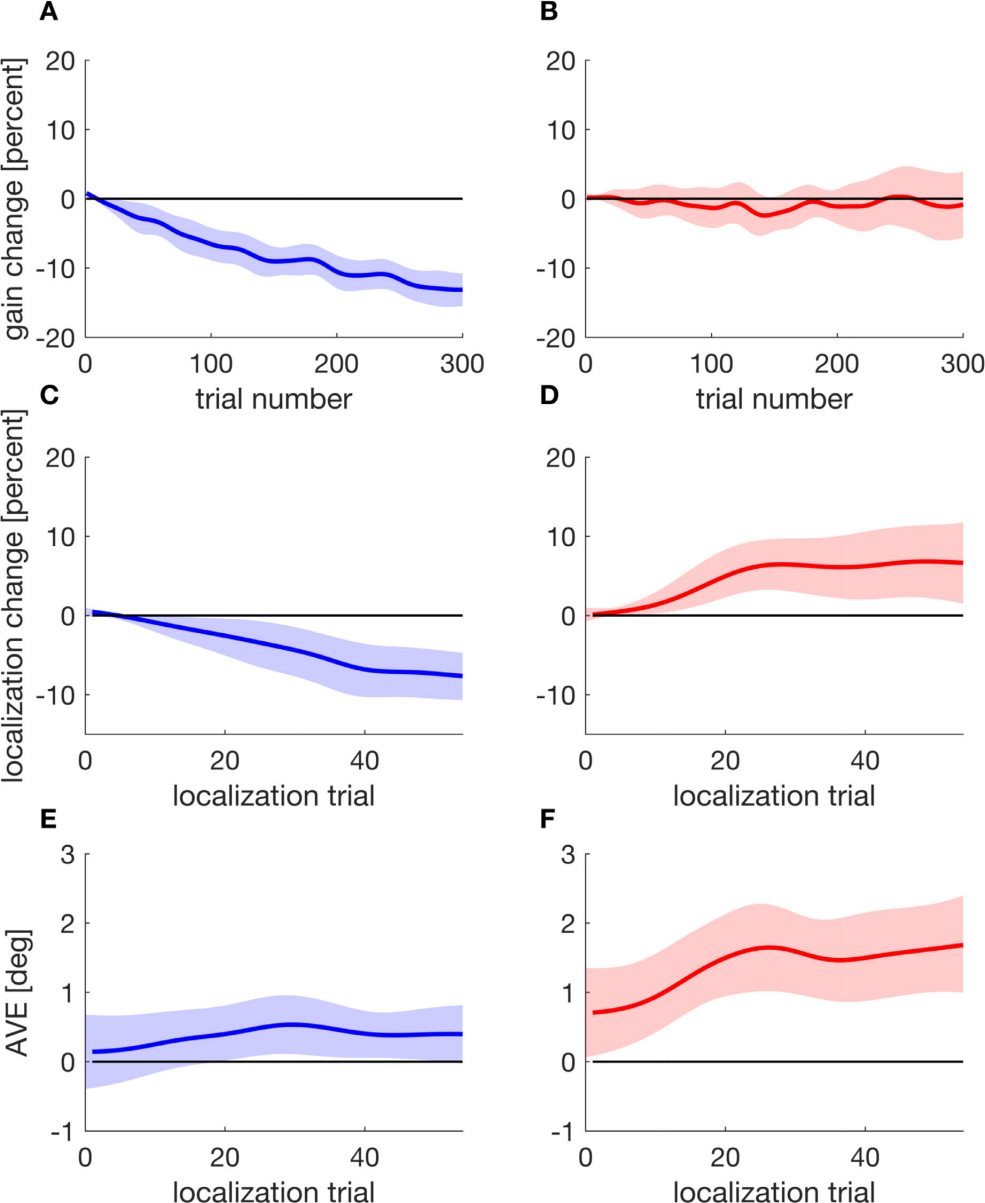
Saccadic gain change, localization change, and apparent visual error as a function of trial, depicted separately for inward (blue) target steps in the left column and for outward (red) target steps in the right column. Shaded areas indicate the 95% confidence intervals.

As there were no designated pre- and post-adaptation trials, we assessed the saccadic gain change and the localization shift as a continuous time series to find out whether and at which time in the experiment our dependent variable deviated from zero. For inward target steps (Figure 7A), gain change deviated significantly from zero from trial 8 onward (*t*_crit_ = 92.140, *t* = 2389.227, *p* < 0.001). For outward target steps, gain change remained around zero throughout the experiment (Figure 7B). These findings are similar to those of Experiment 1 and to our previous study (Heins et al., 2019) as they show successful inhibition of outward adaptation as well as some residual inward adaptation that cannot be inhibited.

Figure 7C,D shows the time course of the localization change. During the course of the experiment, targets were increasingly mislocalized in the respective direction for both inward as well as outward target steps. The SMART analysis indicated, for inward target steps, that localization differed from zero from trial 9 onward (experimental trial 50, *t*_crit_ = 32.573, *t* = 165.890, *p* < 0.001) (Figure 7C). For outward target steps, the change in localization was significant from localization trial 5 onward (experimental trial 27, *t*_crit_ = 35.349, *t* = 167.674, *p* < 0.001) (Figure 7D).

We were particularly interested in the apparent visual error (AVE), since it provides an indication of the difference in adaptation state between the executed saccade and the percept of the target position. Since participants made a saccade toward the target that they subsequently had to localize, the difference should be zero if the saccade and the localization were adapted to the same extent. For inward target steps (Figure 7E), the AVE increased throughout the experiment and exceeded zero significantly from localization trials 19 to 53 (experimental 105 to 291, *t*_crit_ = 33.567, *t* = 88.351, *p* < 0.001). This indicates that the primary saccades undershot the perceived target position. For outward target steps (Figure 7F), the AVE also increased during the experiment and exceeded zero throughout the entire experiment (*t*_crit_ = 32.487, *t* = 270.577, *p* < 0.001). This implies that during inhibition of outward adaptation, participants perceived the target to be increasingly more eccentric than the location to which they actually made the saccade.

The results show that both gain change and localization change developed gradually over time. Residual inward adaptation and the respective shift in perceived position of the saccade target developed in parallel, whereas the localization change following outward target steps occurred even though the saccade gain change remained constant. For this reason, saccade landing point and perceived position of the target stimulus increasingly diverged. These findings indicate that repeated experience of an intrasaccadic target step leads a localization shift in direction of the displacement even when adaptation of saccade amplitude is inhibited.

## Experiment 3—visual reference

The results of Experiment 2 suggest that the shift in localization judgment does not follow adaptive adjustments of saccade amplitude. To further assess the mechanisms driving adaptation of localization, we investigated the influence of explicit post-saccadic feedback about the magnitude and direction of the target displacement on saccade amplitude and localization.

### Method

#### Sample

The sample consisted of 19 participants (12 female) aged between 18 and 46 years (*M* = 26.68, *SD* = 6.13). All participants had normal or corrected-to-normal vision and were right-handed. All participants were recruited from the Institute of Psychology of the University of Muenster and gave their informed consent in written form. Four participants had also participated in either Experiment 1 or Experiment 2. Three participants had to be excluded from data analysis because our inclusion criteria were violated in more than 50% of the trials.

#### Experimental setup and stimuli

**Figure 8.**
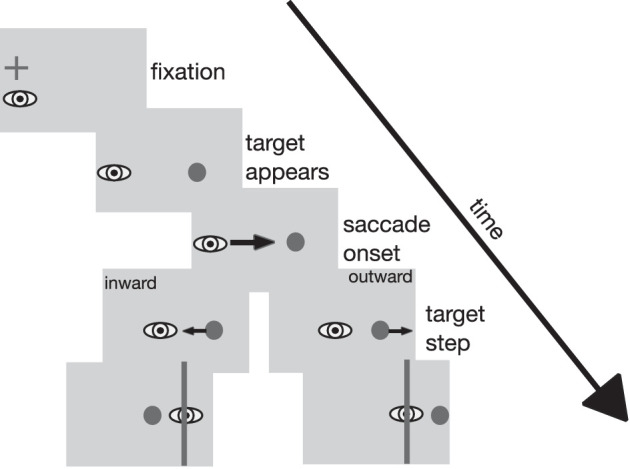
Trial layout for the double-step trials in Experiment 3. Participants first looked at a fixation cross. Then, the fixation cross was turned off and the saccade target appeared 12 deg to the right. Upon saccade onset, the target was shifted either inward or outward, depending on the condition, and simultaneously a vertical line extending from the top to the bottom of the screen was displayed at the initial target position. Stimuli are not drawn true to scale.

The experimental setup of Experiment 3 was the same as in the previous experiments. The stimuli resemble those in Experiment 2. During double-step trials, a vertical gray line extending from the top to the bottom of the screen appeared at the initial horizontal target position as soon as the saccade onset triggered the target step. The joint post-saccadic presentation of the vertical line and the stepped target provided the participants with information about the size and direction of the target displacement as well as their saccade accuracy (Figure 8). The localization trials corresponded to those of Experiment 2. Participants had to saccade toward a target stimulus, which disappeared upon saccade onset and indicate its position on the screen after saccade landing. Unlike in Experiment 2, the cursor appeared with a 750-msec delay after the target stimulus had been turned off to prevent distraction from the saccade target even more reliably.

The experiment consisted of two recording sessions per participant, outward inhibition and inward inhibition, each lasting approximately 25 min and comprising 300 trials. These consisted of 246 double-step trials and 54 localization trials. The localization trials were arranged in the same manner as in Experiment 2, and every 60 trials, the experiment paused and the audio file with the instruction was played.

#### Data analysis

The data analysis resembles that of Experiment 2. Exclusion criteria for saccades and localization judgments were the same as in the previous experiments. A total of 13.83% of saccades and 3.99% of the localization judgments had to be discarded. Gain change, localization change, and AVE were calculated analogous to Experiment 2.

#### Results

**Figure 9.**
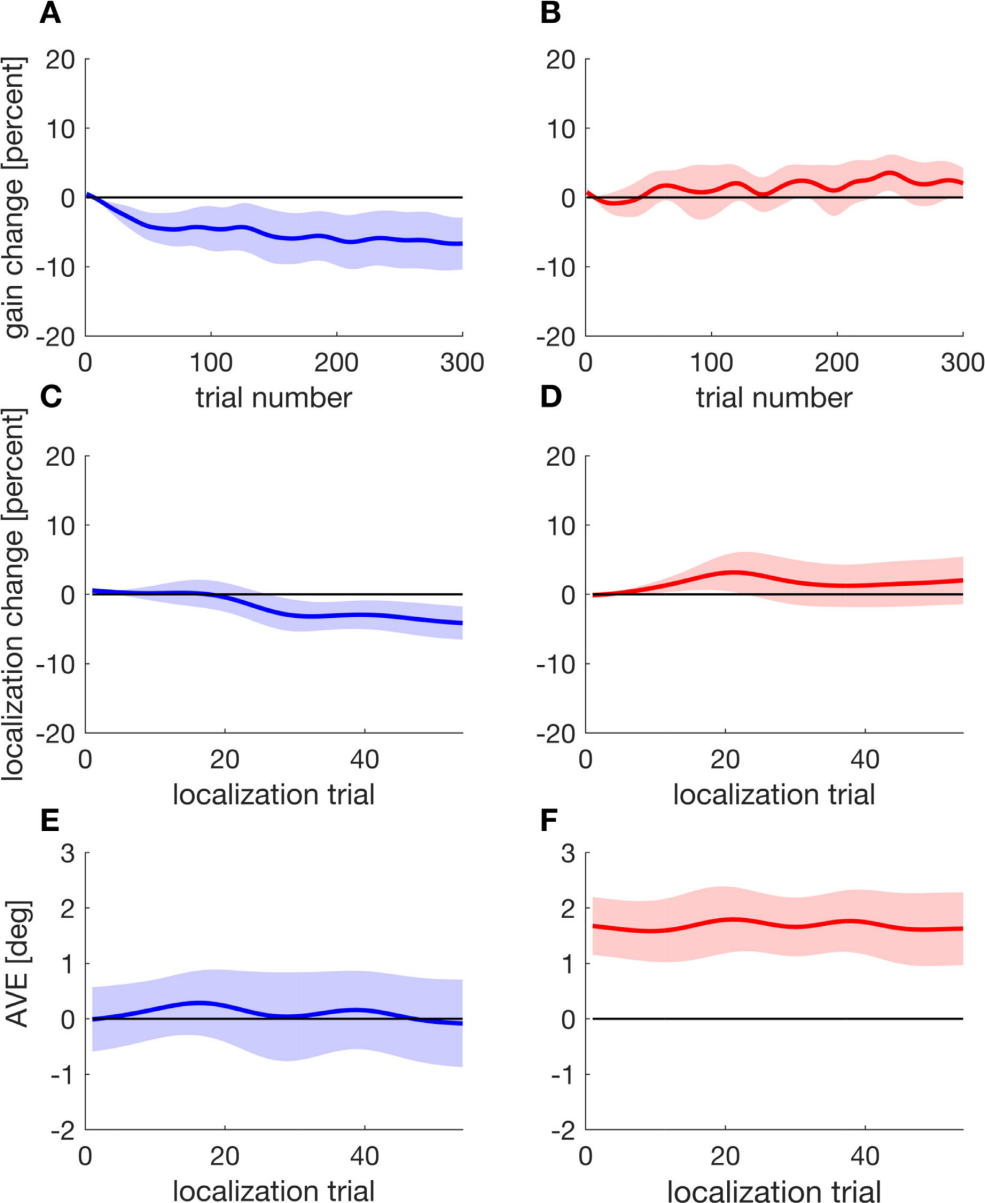
Saccadic gain change, localization change, and apparent visual error as a function of trial, depicted separately for inward (blue) and outward (red) target steps. Shaded areas indicate the 95% confidence intervals.

For inward target steps, saccadic gain change (Figure 9A) decreased throughout the experiment and was significantly below zero from trial 9 onward (*t*_crit_ = 95.413, *t* = 1003.883, *p* < 0.001). For outward target steps (Figure 9B), saccadic gain change remained around zero throughout the experiment. The localization shifted increasingly in direction of the target displacement for inward target steps. From localization trial 27 (experimental trial 152) onward, the localization change fell below zero (*t*_crit_ = 32.041, *t* = 88.277, *p* < 0.001) (Figure 9C). For outward target steps (Figure 9D), the localization judgment shifted significantly in outward direction between localization trials 8 to 22 (experimental trials 42 to 120, *t*_crit_ = 34.398, *t* = 39.641, *p* = 0.031). Figures 9E,F shows the time course of the AVE. For inward target steps, the AVE remained constantly around zero, indicating that the adaptation state of the executed saccade and the localization judgment did not differ. For outward target steps, the AVE was above zero throughout the whole experiment (*t*_crit_ = 32.636, *t* = 334.897, *p* < 0.001), indicating a mismatch between the localization judgment of the saccade target and the executed saccade itself. Thus, providing a post-saccadic visual reference to the initial target position did not prevent a shift of perceived target location in the direction of the target step altogether, but the explicit information about the magnitude and direction of the target step provided by the post-saccadic visual reference seems to have strengthened the ability to inhibit. To verify this impression, we calculated a measure of late gain change from the last 20 trials and a measure of late localization change from the last 10 localization judgments and compared the magnitude of adaptation for both inward and outward target steps between Experiment 2 and Experiment 3. We found that saccadic gain change following inward target steps was indeed smaller in Experiment 3 than in Experiment 2 (*t*(15) = –4.565, *p* < 0.001, one-sided unpaired *t* test). Thus, for inward target displacement, the inhibition of amplitude adaptation worked better when a visual reference to the initial target position was provided. For outward target steps, no such difference occurred between Experiment 3 and Experiment 2 (*t*(15) = –1.739, *p* = 0.952, one-sided unpaired *t* test; *BF*_01_ = 0.962 [1.119, 1.379]), which is not surprising given that saccadic gain change already remained around zero in Experiment 2. The localization change was not significantly attenuated when a visual reference was provided, neither for inward (*t*(15) = –1.659, *p* = 0.054, one-sided unpaired *t* test; *BF*_01_ = 1.059 [1.247, 1.550]) nor for outward target displacement (*t*(15) = 1.939, *p* = 0.093, one-sided unpaired *t* test; *BF*_01_ = 0.735 [0.833, 1.013]).

## Experiment 4—overlap saccades

Experiment 3 examined the influence of post-saccadic information about the size and direction of the target step on the ability to inhibit adaptive changes to saccade amplitude and localization. In Experiment 4, we aimed at investigating the influence of extended pre-saccadic information. We measured saccade amplitude and localization during overlap saccades. During localization, target-off, and double-step trials, an initial valid fixation was followed by joint presentation of the fixation cross and the saccade target. The simultaneous presentation of fixation cross and saccade target, and the resulting increase in preview duration of the saccade target compared to regular reactive saccades, has been shown to lead to weaker saccadic suppression of displacement, thus allowing for a better comparison between pre- and post-saccadic target positions (Zimmermann, Morrone, & Burr, 2013). We hypothesized this would lead to a more accurate comparison between pre- and post-saccadic target positions and, accordingly, that the post-saccadic error would be attributed to the actual position change of the target rather than to a motor error. We expected that this would further attenuate adaptation of saccade amplitude and would also reduce the localization shift, as the association between pre- and post-saccadic visual information becomes weaker. As in Experiment 1, the changes in localization and in saccade amplitude were assessed.

### Method

#### Sample

The sample consisted of 18 participants (10 female) aged between 18 and 46 years (*M* = 25.42, *SD* = 6.55). All participants had normal or corrected-to-normal vision and were right-handed. All participants were recruited from the Institute of Psychology of the University of Muenster and gave their informed consent in written form. Fourteen participants had participated in one of the previous experiments. Two participants had to be excluded from data analysis because our inclusion criteria were violated in more than 50% of the trials.

#### Experimental setup and stimuli

**Figure 10.**
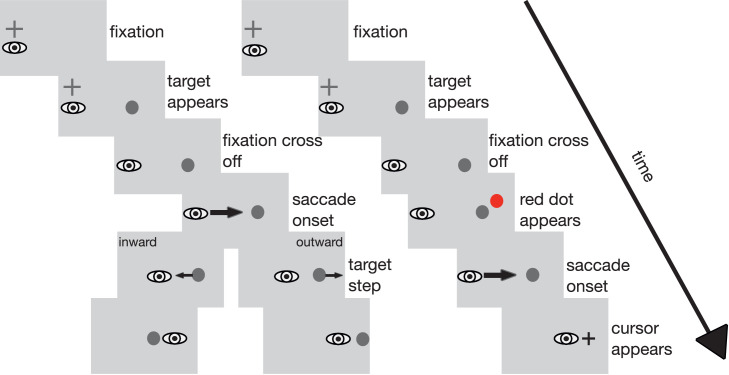
Trial layout for double-step trials (left) with inward and outward target steps and for localization trials (right) in Experiment 4. In the double-step trials, participants first looked at a fixation cross. The saccade target appeared 12 deg to the right. The participants had to maintain fixation on the fixation cross until it was removed from the screen. This served as the go-signal for the saccade. Upon saccade onset, the target was stepped, depending on the condition, either in the inward or the outward direction, leading to a post-saccadic error after saccade landing. In the localization trials, the participants also first looked at the fixation cross. The saccade target appeared 12 deg to the right and participants had to keep gaze at the fixation cross until it was removed. Fifty milliseconds later (i.e., during the saccade latency period), a red probe dot was displayed for two frames (27 msec). After saccade onset, the saccade target was turned off and the cursor appeared. After the saccade, participants indicated the perceived position of the red probe dot with the cursor. Stimuli are not drawn true to scale.

The experimental setup of Experiment 4 was the same as in the previous experiments. The stimuli resemble those in Experiment 1 except for an overlap in presentation time for the fixation cross and the target stimulus. Thus, the sequence of events is slightly different (Figure 10). At the start of each trial, valid fixation of the fixation cross was ensured as in the previous experiments. Following detection of valid fixation, the target stimulus was displayed 12 deg to the right of the fixation cross. Fixation cross and target stimulus were presented together for 500 msec. During this time span, the participants were to hold fixation on the fixation cross. If their eye left the fixation window, a sine tone indicated that fixation was broken and the trial was aborted and repeated. After valid fixation throughout the joint presentation time of fixation cross and target stimulus had been confirmed, the fixation cross was turned off. This served as the go-signal for the saccade toward the target stimulus. Subsequent events depended on the type of trial. As in Experiment 1, double-step trials, localization trials, and target-off trials were used. During the double-step trials, the target was stepped as soon as the saccade was detected. During target-off trials, the target was turned off as soon as the saccade had been detected. During the localization trials, a red probe dot appeared for two video frames (27 msec) starting 50 msec after the fixation cross was turned off during saccade preparation time. The target stimulus disappeared as soon as saccade onset had been detected. Unlike in Experiment 1, the cursor appeared with a delay of 750 msec after saccade onset to avoid confusion with either the saccade target or the localization probe even more reliably. The experiment consisted of two recording sessions per participant, outward inhibition and inward inhibition, each lasting approximately 20 min. The different trial types were arranged in the same manner as in Experiment 1. As in the previous experiments, every 60 trials, the experiment paused and the audio file containing the instruction was played.

#### Data analysis

The data analysis resembles that of Experiment 1. Exclusion criteria for localization judgments and saccades were the same as in the previous experiments, with the exception of primary saccade latencies. Since overlap saccades are voluntary eye movements, saccades with a latency of less than 100 msec as well as outliers were removed from the data analysis. Outliers were defined as saccades with latencies that were more than 3 median absolute deviations away from the median latency of the respective participant. A total of 18.56% of saccades and 8.05% of the localization judgments had to be discarded. Gain change and localization change were calculated analogous to Experiment 1.

#### Results

**Figure 11.**
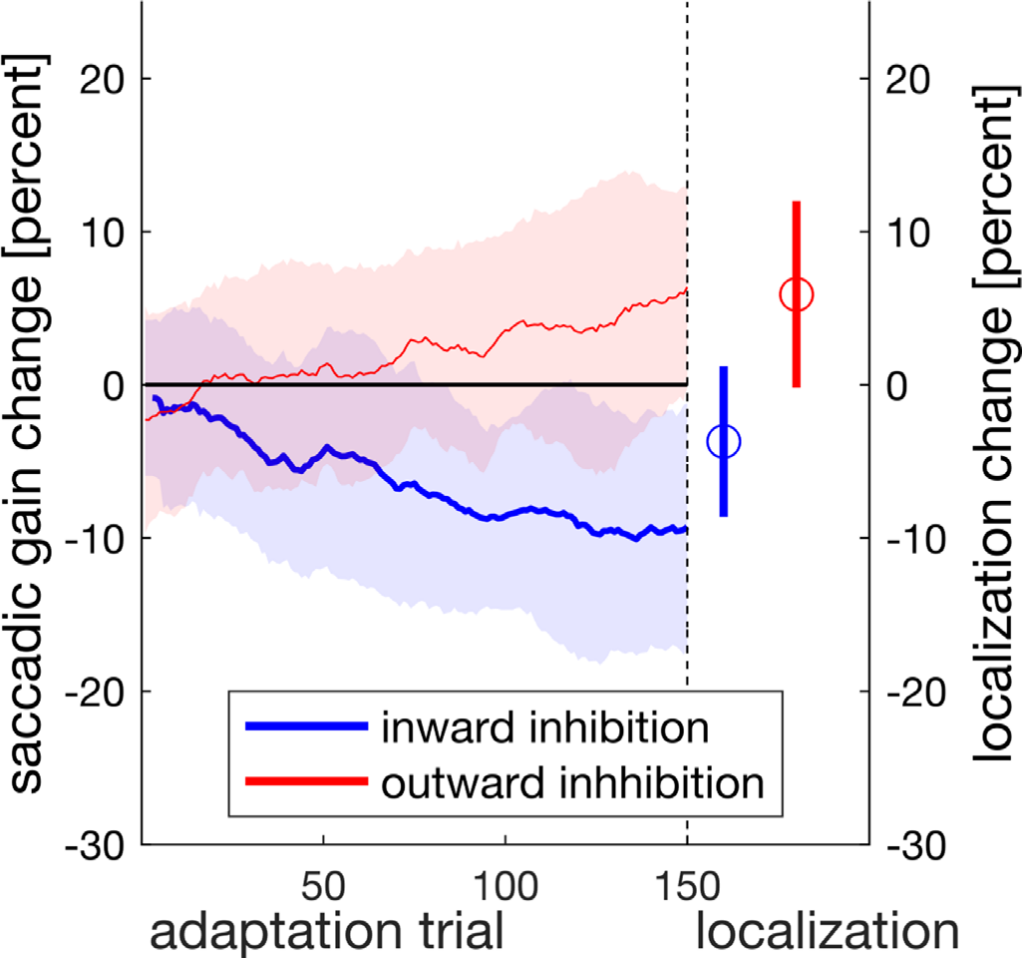
Moving average (window size 20 trial) of the saccadic gain change during the double-step procedure and mean localization change during the subsequent localization trials for inward (blue) and outward (red) target steps. The shaded areas as well as the vertical bars indicate standard deviations.

The time courses for saccadic gain change following the inhibition instruction for both inward and outward target steps resemble that of Experiment 1 (Figure 11). Mean saccadic gain change during late adaptation (trials 171:190) was below zero (*M* = –9.47%, *SD* = 7.70%; *t*(15) = –4.916, *p* < 0.001) following inward target steps. For outward target steps, gain change exceeded zero (*M* = 5.53%, *SD* = 7.62%; *t*(15) = 2.900, *p* = 0.007, one-sided *t* test). We also analyzed saccadic gain change during post-adaptation trials (211:230) that followed the localization trials (191:210). Following inward target steps, gain change was below zero (*M* = – 9.12%, *SD* = 5.02%; *t*(15) = –7.271, *p* < 0.001, one-sided *t* test), and for outward target steps, saccadic gain change exceeded zero (*M* = 5.33%, *SD* = 5.93%; *p* = 0.006, one-sided Wilcoxon signed-rank test).

The localization of the probe following double-step trials with inward target displacement shifted significantly in the direction of the target step (*M* = –4.71%, *SD* = 4.91%; *t*(15) = –3.007, *p* = 0.004, one-sided *t* test). The same applies to the localization change following outward target steps (*M* = 5.90%, *SD* = 6.10%; *t*(15) = 3.872, *p* = 0.002, one-sided *t* test).

Judging from these results, saccadic gain change following outward target steps was no longer inhibited completely, as it exceeded zero both during late- and post-adaptation trials. Yet, the magnitude of saccadic gain change in this experiment appears rather similar to that of Experiment 1. In fact, there was no significant difference between this experiment and Experiment 1 in gain change for inward target steps, neither during late- nor during post-adaptation trials (late: *t*(15) = 0.595, *p* = 0.561, two-sided unpaired *t* test, *BF*_01_ = 3.344 [4.484, 6.135]; post: *t*(15) = 0.310, *p* = 0.761, *BF*_01_ = 3.745 [5.051, 6.944]). The same applied to outward target steps (late: *t*(15) = –0.953, *p* = 0.356, two-sided unpaired *t* test, *BF*_01_ *=* 2.639 [3.460, 4.673]; post: *t*(15) = –0.538, *p* = 0.599, two-sided unpaired *t* test, *BF*_01_ *=* 3.448 [4.608, 6.329]). Localization change following overlap saccades was also not significantly different from that following reactive saccades (inward: *t*(15) = –1.803, *p* = 0.092, two-sided unpaired *t* test; *BF*_01_ = 1.053 [1.290, 1.664]; outward: *t*(15) = 0.225, *p* = 0.825, two-sided unpaired *t* test, *BF*_01_ = 3.831 [5.181, 7.092]). We conclude that the ability to inhibit adaptive changes to saccade motor performance is not different for overlap compared to reactive saccades and that the increase in preparation time that is available for overlap saccades did not affect the ability to inhibit adaptive changes to the saccade itself or the localization judgment.

**Figure 12.**
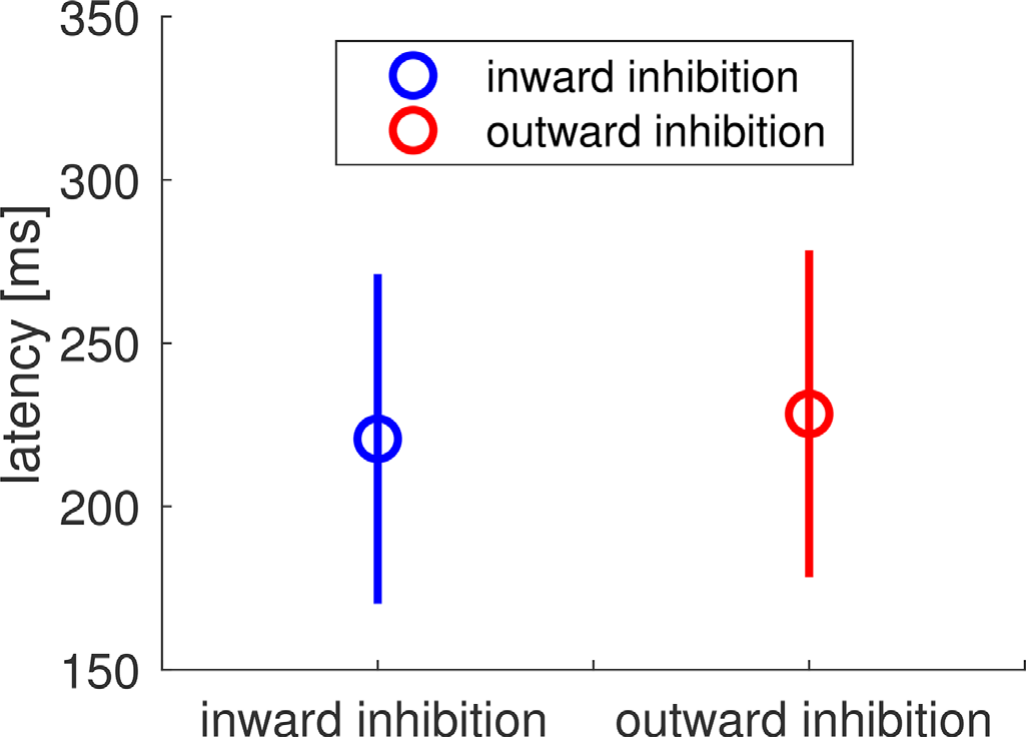
Mean saccadic latency for inward (blue) and outward (red) target steps, depicted for the late-adaptation trials. The bars indicate standard deviations.

The average saccadic latency during the late-adaptation trials (Figure 12) did not differ significantly for outward (*M* = 228.36 msec, *SD* = 50.03 msec) and inward target steps (*M* = 220.70 msec, *SD* = 50.52 msec; *t*(15) = 0.619, *p* = 0.545, two-sided *t* test). The same applies to saccadic latency during post-adaptation trials. There was no significant difference in saccadic latency between inward and outward target displacement (outward: *M* = 213.08 msec, *SD* = 44.97 msec; inward: *M* = 216.62 msec, *SD* = 50.82 ms; *t*(15) = –0.231, *p* = 0.820, two-sided *t* test). The absence of a significant difference in saccadic latency between the inward and outward inhibition condition is not surprising, even though the inhibition instruction typically leads to an increase in saccade latency for reactive saccades in the outward condition. In the overlap condition, participants had ample time to plan their saccade before the go-signal due to the joint presentation time of fixation cross and target stimulus. Possible differences in saccade preparation time that exist between the two conditions might therefore not be noticeable.

## Experiment 5—fatigue

In all our previous experiments, we observed stronger residual adaptation following inward than following outward target steps. To ensure that this was in fact due to a true difference in inhibitory ability, we assessed the influence of repeated execution of a stereotyped saccade on saccade amplitude and localization over time. Therefore, in Experiment 5, regular saccade trials were randomly interspersed with target-off localization trials. Saccade amplitude and localization were measured throughout the experiment.

### Method

#### Sample

The sample consisted of 8 participants (4 female) aged between 23 and 53 years (*M* = 32.5, *SD* = 11.23). All participants had normal or corrected-to-normal vision and were right-handed. All participants were recruited from the Institute of Psychology of the University of Muenster and gave their informed consent in written form. Six participants had participated in one of the previous experiments.

#### Experimental setup and stimuli

The experimental setup of Experiment 5 was the same as in the previous experiments. The localization trials correspond to those of Experiments 2 and 3. The participants had to saccade toward a target stimulus that disappeared upon saccade onset and indicate its position on the screen after saccade landing. Unlike in Experiment 2, the cursor appeared with a 750-msec delay after the target stimulus had been turned off to prevent distraction from the saccade target even more reliably. During regular saccade trials, participants had to saccade toward a target stimulus that remained on screen for another 500 to 1000 msec after saccade detection. Participants thus received visual feedback after saccade landing in the same manner as during the double-step trials in the previous experiments.

One session per participant was recorded, each lasting approximately 25 min and comprising 300 trials. These consisted of 246 target-on trials and 54 localization trials. The localization trials were randomly arranged with the condition that a minimum of three and a maximum of six target-on trials were run in between.

As in the previous experiments, this experiment also paused every 60 trials for 25 sec. We inserted this pause to ensure the comparability of the sequence of events between the different experiments. No verbal instruction was played during the break in order to not distort the effect of any natural fatigue that might occur.

#### Data analysis

The data analysis resembles that of Experiments 2 and 3. Exclusion criteria for saccades and localization judgments were the same as in the previous experiments. Overall, 11.63% of saccades and 3.70% of localization judgments were discarded. Gain change, localization change, and AVE were calculated analogous to Experiment 2.

#### Results and discussion

**Figure 13.**
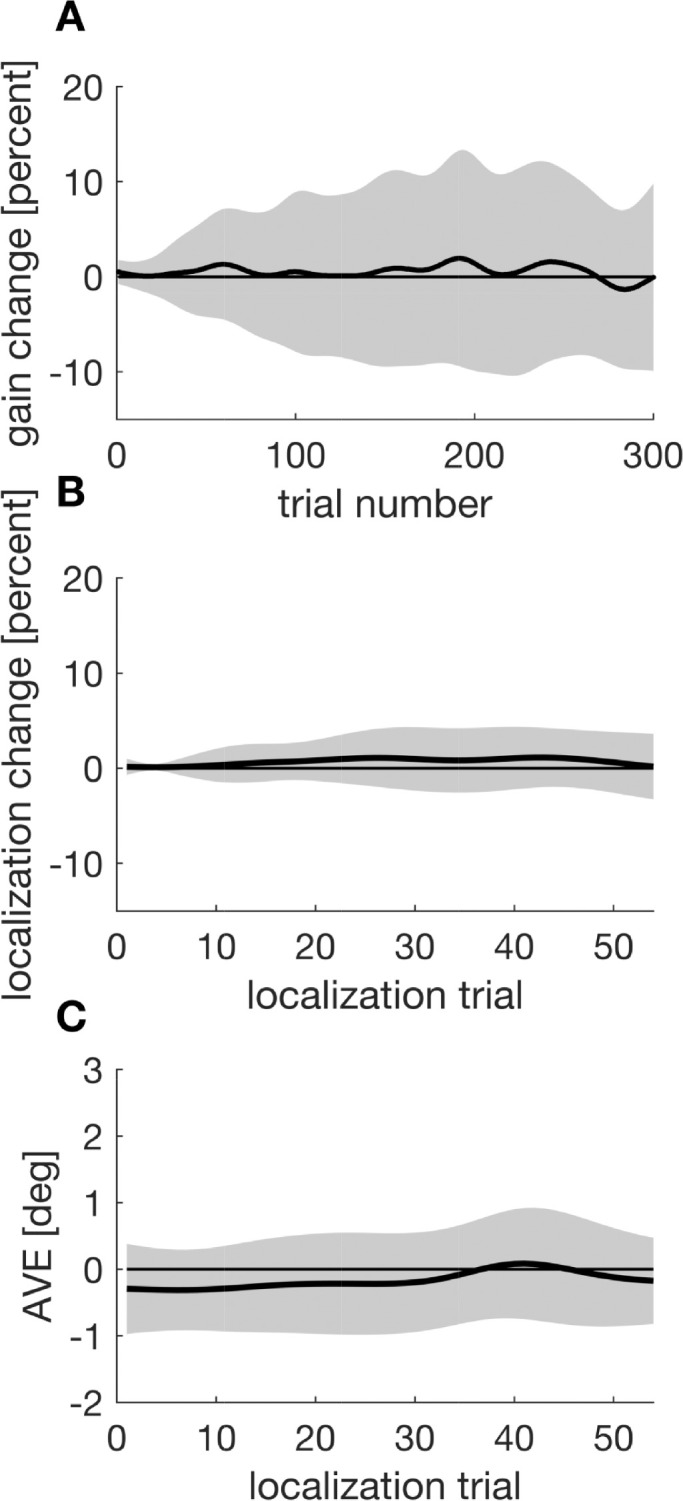
Saccadic gain change, localization change, and apparent visual error as a function of trial. Shaded areas indicate the 95% confidence intervals.

Saccadic gain change (Figure 13A) remained around zero throughout the experiment. The same applies to saccade localization change (Figure 13B) and the AVE (Figure 13C). This implies that the saccade target was localized at the position to which the saccade was made. Thus, repeated saccade execution toward a target stimulus had no effect on gain change or localization in our experimental paradigm. Accordingly, the residual inward adaptation following inward target steps, which occurs in the inhibition condition, reflects actual adaptation to the visual error signal. Likewise, the lack in significant changes to saccadic gain following outward inhibition cannot be attributed to fatigue but rather reflects stronger inhibition abilities.

## General discussion

When the target of a saccade is consistently displaced during the saccade, human observers gradually adapt their saccade amplitude to better reach the target. This saccadic adaptation is associated with a shift of the perceived position of objects near the saccade target in the direction of the target displacement. The modification localization may be a consequence of adaptive changes to saccade amplitude or it may be a generator of adaptive changes to saccade amplitude. In the present study, we showed that the localization shifts occur even when adaptive changes to saccade amplitude are actively inhibited. The shift of perceived spatial location did not only affect localization probes in the vicinity of the saccade target flashed during saccade preparation but also extended to the saccade target itself. These results show that the localization shift develops even when no or only small changes occur in the amplitudes of the saccades that are executed during the adaptation procedure.

Experiment 1 compared the instruction to direct saccades to the initial target and to continue to look at that position with the instruction to follow the target to its final position. The magnitude of the shift in perceptual localization was equally strong for the inhibition and the adaptation instruction. Thus, the localization shift developed even as changes to the saccade amplitude were inhibited during the adaptation procedure. This implies that the inhibition instruction affected primarily the amplitude of the executed saccade. While adaptive changes to the saccadic amplitude were attenuated, the localization shift developed throughout the course of the experiment. In Experiment 2, we extended those findings to the apparent location of the saccade target itself and studied the course of adaptive changes to saccadic amplitude and target localization. Both gain change and localization change developed gradually over time. Residual inward adaptation and the respective shift in perceived position of the saccade target developed in parallel. For outward target steps, adaptive changes to the localization judgment developed while saccadic gain change remained around zero. Consequently, the saccade landing point and the perceived position of the target stimulus increasingly diverged during the course of the experiment. In Experiment 3, explicit information about the magnitude and direction of the target step was presented during the adaptation phase. The adaptive changes to saccadic amplitude following the inhibition instruction were less pronounced than in Experiment 2, during which no visual reference was provided. Thus, a visual reference to the initial target position strengthened the ability to perform saccades aimed toward the initial target position. Since the post-saccadic information about the initial target position as well as the target step did not rule out adaptation entirely, we wondered if extended pre-saccadic information would yield another pattern of results. Thus, in Experiment 4, the influence of increased preparation time on the ability to inhibit adaptive changes was studied using overlap saccades. Both adaptive changes to saccade amplitude and localization judgments remained unaffected. Since our results consistently showed more residual adaptation following inward than outward target steps, we conducted Experiment 5 and assessed whether repeated execution of a stereotyped saccade toward a target stimulus would produce a systematic effect on saccadic gain change or localization. This was not the case. Neither saccade amplitude nor localization changed throughout the experiment. Those results suggest that the residual adaptation after inward target steps reflects the actual adaptive changes induced by the intrasaccadic target step, and weaker changes following outward target steps are in fact the result of stronger inhibition abilities.

Taken together, the results of this study support and extend the findings of our previous study on inhibition of saccadic adaptation. Inhibition of saccadic adaptation is thought to involve both suppression of a reflexive, adapted saccade and preserving the movement vector specifying a saccade toward the initial target position (Heins et al., 2019). The current study supports that a reflexive saccade toward the perceived target position is suppressed and a saccade that better matches the instruction is executed. During the adaptation procedure, this inhibitory mechanism presents in attenuated or completely inhibited adaptive changes to the saccade amplitude as well as in a significant increase in saccade latency for outward target steps for which inhibition of adaptive changes to the saccade amplitude is more complete.

Our inhibition instruction explicitly disclosed to participants that the target might not be in the same position after the saccade as it was before. We explicitly instructed our participants either to look at the first target location and remain there irrespective of any further change of the target, or to follow the target toward its final position. Previous studies of saccade adaptation have typically not distinguished between these possibilities, since small target displacements usually are not noticed (Bridgeman, Hendry, & Stark, 1975; Deubel, Wolf, & Hauske, 1986; Stark, Kong, Schwartz, & Hendry, 1976). Bahcall and Kowler (1999, 2000) used an instruction similar to ours and encouraged participants to make precise primary saccades to the location where the target was seen before the saccade and not to be concerned with any post-saccadic errors they might notice. They found mislocalization in the direction of the target shift, consistent with our findings, but also that saccade amplitude adapted, which was not the case for outward target shifts in our study. The difference in the pattern of results may have several reasons. First, our target step might have been easier to detect. On average, the step size in our study was 30% of the required saccade amplitude while it was only 20% in the work of Bahcall and Kowler (1999, 2000). Furthermore, the experiments of Bahcall and Kowler were conducted in darkness. In our study, visual reference cues such as screen borders might have further facilitated the detection of the target displacement with respect to the initial target position, possibly making it easier for our participants to maintain a stable saccade gain. Despite these differences, the localization results of our study appear consistent with those of Bahcall and Kowler. In both our study and the work of Bahcall and Kowler (1999, 2000), a localization shift occurred following instructions to look at the initial target position and disregard any further target movement. This localization shift seems to reflect the neural recalibration after repeated exposure to the double-step task. Moreover, in our study, it occurs with the same magnitude for both instructions. In line with earlier reports (Bahcall & Kowler, 1999, 2000; Collins & Wallman, 2012), this gradual neural recalibration, which we found in Experiments 2, 3, and 4, cannot be attributed to post-saccadic retinal error. If retinal error had driven the localization shift, we would have observed a stronger localization shift following the inhibition instruction than following the adaptation instruction because of the large and persisting uncorrected errors. Second, and also in line with earlier reports (Wallman & Fuchs, 1998), the neural recalibration does not depend on corrective saccades following the primary saccades. The magnitude of the localization shift is the same for the inhibition and adaptation instruction, whereas secondary saccades occur less frequently following the inhibition instruction (Heins et al., 2019). It thus appears most likely that the efference copy of the saccade is involved in the process of neural recalibration (Bahcall & Kowler, 1999, 2000; Cavanaugh, Berman, Joiner, & Wurtz, 2016; Collins & Wallman, 2012; Masselink & Lappe, 2021). In addition, our results indicate that the amplitude of the executed saccade does not always reflect this adaptation state, because the localization shift is equally strong for both instructions, whereas the adaptive changes to the saccade amplitude are not.

The localization shift after inhibition of adaptation may also occur without changes to the amplitude of the executed saccades if the repeated intrasaccadic target displacement triggers learning of a new association between pre- and post-saccadic target positions. This appears reminiscent of transsaccadic perceptual calibration of target features such as size (Bosco, Lappe, & Fattori, 2015; Valsecchi, Cassanello, Herwig, Rolfs, & Gegenfurtner, 2020; Valsecchi & Gegenfurtner, 2016) or shape (Herwig & Schneider, 2014; Herwig, Weiß, & Schneider, 2015; Köller, Poth, & Herwig, 2020; Paeye, Collins, Cavanagh, & Herwig, 2018). These studies showed that associations are learned between pre-saccadic peripheral and post-saccadic foveal input and used to calibrate peripheral object perception. New associations can be formed through systematic intrasaccadic manipulation of the post-saccadic foveal input (e.g., through an object that systematically alters its size or shape during the saccade). Perception of the pre-saccadic peripheral view of an object is then biased toward the high-resolution post-saccadic foveal view. The learning process itself may be of a general associative type and not rely on the occurrence of a saccade (Bosco, Rifai, Wahl, Fattori, & Lappe, 2020; Paeye et al., 2018; Valsecchi & Gegenfurtner, 2016), but in everyday life, it will most likely occur in relation to transsaccadic feedback. It is tempting to assume that, in our study, participants might have learned new associations between the pre- and the post-saccadic location of the target area, which might have led to a biased spatial perception of any visual stimuli in the vicinity of the saccade target in direction of the post-saccadic information (i.e., displaced in the direction of the target step). Thus, the development of the localization shift in the absence of adaptation of the motor command could partially rely on perceptual calibration of visual space perception in response to systematic intrasaccadic manipulation of the pre-saccadic peripheral and post-saccadic foveal input. However, unlike changes in target features, which in contrast to saccadic adaptation can generalize across hemifields (Valsecchi et al., 2020), a learning process involving pre- and post-saccadic position cannot solely rely on retinal input but requires also information about the change in retinal direction obtained, for example, from efference copy signals (Cavanaugh et al., 2016). Thus, any such learning process would have to take these signals into account. Perhaps this induces the spatial selectivity that is observed for the shift in perceived target location.

Moreover, our study provides further evidence for the target selectivity of saccadic adaptation as well as its implicit nature. When we provided our participants with a post-saccadic visual reference to the initial target position, a shift in localization judgment in the direction of the target step still developed, indicating that adaptation of localization was not prevented even when participants were given visual information about the target step. At first sight, this might seem like a surprising finding, given that this visual feedback informs the participant not only about the magnitude and direction of the target displacement, but also about the accuracy of their own saccade toward the initial target position. Yet, sensorimotor adaptation happens implicitly due to a mismatch between expected and actual sensory consequences of the movement outcome, and the application of strategies has been shown to be overruled by sensory feedback (Mazzoni & Krakauer, 2006). Since saccade adaptation involves pre-saccadic target selection and is then driven by visual errors induced by this target only (Madelain, Harwood, Herman, & Wallman, 2010), our participants experienced the same error signal as in our other experiments despite the visual reference object.

The data of the present experiment, like many other studies before (Ethier, Zee, & Shadmehr, 2008; Miller, Anstis, & Templeton, 1981; Panouillères et al., 2009; Pélisson et al., 2010; Straube & Deubel, 1995; Straube, Fuchs, Usher, & Robinson, 1997), showed differences between inward and outward target steps. The finding that inhibition for outward target steps was more successful in preventing a change in saccadic gain than for inward target steps is consistent with the predominant finding that different mechanisms underlie inward and outward adaptation. Outward adaptation is weaker and less stable than inward adaptation (Miller et al., 1981; Straube & Deubel, 1995; Straube et al., 1997), which might, along with the natural hypometria of the saccadic system (Becker, 1989), have contributed to a lower adaptation level in the outward inhibition than in the inward inhibition condition. Moreover, it has been suggested that inward adaptation relies more on internal adjustment of the ongoing motor performance, while outward adaptation relies more on a remapping of the target position (Ethier et al., 2008; Hernandez, Levitan, Banks, & Schor, 2008; Panouillères et al., 2009; Semmlow, Gauthier, & Vercher, 1989; Zimmermann & Lappe, 2010, 2016). Our finding that the residual adaptation observed for inward target steps reflects actual adaptive changes and that the lack of significant amplitude adaptation following outward target steps is due to stronger inhibitory abilities adds to the list of differences between inward and outward adaptation.

Perhaps these differences may be reconciled by the observation of further differences between saccades in inward and outward inhibition conditions. In the present study as well as in our prior study (Heins et al., 2019), reactive saccades in the outward inhibition condition showed longer latencies than in the outward adaptation condition. This was not the case for inward target steps. We proposed earlier that the longer latency might reflect the inhibitory mechanism that suppresses a reflexive saccade to the newly learned target location and a replanning of a saccade toward a less eccentric location. If the saccadic system would initially prepare a visually guided saccade to the perceived target location and then abort and replan a shorter saccade, the increase in saccadic latency might reflect this replanning. Findings from the post-adaptation target-off trials in Experiment 1 support this view. When the intrasaccadic displacement of the saccade target was no longer applied, the effect of instruction on saccadic latency was no longer substantial and a significant gain change emerged. In addition, the correlation between gain change and localization change following the inhibition instruction was significant during the post-adaptation but not the late-adaptation trials, indicating that the adaptation state of the motor program of the saccade and the localization were related once inhibition was discontinued. It seemed that when participants noticed that the intrasaccadic manipulation was removed, they no longer actively aborted their reflexive saccades in favor of executing a 12-deg saccade to the initial target position, and thus the adapted state of the saccade motor program, latently acquired during the adaptation procedure, became manifest. This resembles the latent learning observed in a recent study (Heins & Lappe, 2022) in which participants had to make an eye movement toward a specified object within an object array. Participants had to keep saccade gain constant in order to achieve the task goal (i.e., to foveate the target object), although the object array was shifted against saccade direction during the saccade. Participants thus had to inhibit adaptation to a position error to fulfill the task. They managed to actively control their oculomotor behavior and maintain a stable saccade gain for as long as the intrasaccadic manipulation was applied. However, when the intrasaccadic manipulation and the post-saccadic feedback were removed, participants showed a significant aftereffect (i.e., there was significant gain change in direction of the array shift). It seemed that the repeated intrasaccadic manipulation led to latent learning that was masked by strategic oculomotor behavior until the intrasaccadic displacement was no longer applied and participants deemed it no longer necessary. The results of our current study are consistent with this finding in that in the outward inhibition condition of Experiment 1, saccade gain change was not significantly different from zero during the late-adaptation trials, during which the target was still displaced during the saccade target steps but exceeded zero during the post-adaptation trials without the target step. We suggest that localization of the target adapted because of the continuing intrasaccadic manipulation during the adaptation phase, as shown by the typical adaptation-induced localization shift, and that active, volitional control was required to suppress the execution of a saccade to the perceived target position in order to comply with the instruction. In this view, our results do not argue against adaptive changes to the motor command of the saccade developing as a consequence of the intrasaccadic target steps. The saccade motor command and the perceived target position may be adapted at a common adaptation site in the brain, downstream from which voluntary control is exerted upon the eye movement only, resulting in inhibition of amplitude adaptation and intact adaptation of the perceived target position.

## Conclusion

Our findings indicate that adaptation of localization evolves also when adaptive changes to the amplitude of the saccades executed are inhibited. This suggests that either the intrasaccadic manipulation triggers the localization shift or the saccade amplitude can adapt latently, together with perceived location, but voluntary control is exerted on the execution of the eye movement only. Either case would result in the apparent divergence of the adaptation states of saccade amplitude and perceived location when amplitude adaptation is inhibited.
